# Reconstruction of skeletal movement using skin markers: comparative assessment of bone pose estimators

**DOI:** 10.1186/1743-0003-3-7

**Published:** 2006-03-23

**Authors:** Andrea Cereatti, Ugo Della Croce, Aurelio Cappozzo

**Affiliations:** 1 Department of Human Movement and Sport Sciences, Istituto Universitario di Scienze Motorie, Rome, Italy; 2 Department of Biomedical Sciences, University of Sassari, Sassari, Italy; 3 Centro di Cura e Riabilitazione Santa Maria Bambina, Oristano, Italy

## Abstract

**Background:**

The assessment of the accuracy of the pose estimation of human bones and consequent joint kinematics is of primary relevance in human movement analysis. This study evaluated the performance of selected pose estimators in reducing the effects of instrumental errors, soft tissue artifacts and anatomical landmark mislocations occurring at the thigh on the determination of the knee kinematics.

**Methods:**

The pattern of a typical knee flexion-extension during a gait cycle was fed into a knee model which generated a six-components knee kinematics and relevant marker trajectories. The marker trajectories were corrupted with both instrumental noise and soft tissue artifacts. Two different cluster configurations (4 and 12-marker cluster) were investigated. Four selected pose estimators, a Geometrical method, a SVD-based method, and the Pointer Cluster Technique in the optimized and non optimized version, were analyzed. The estimated knee kinematics were compared to the nominal kinematics in order to evaluate the accuracy of the selected pose estimators.

**Results:**

Results have shown that optimal pose estimators perform better than traditional geometric pose estimators when soft tissue artifacts are present. The use of redundant markers improved in some cases the estimation of the dynamics of the kinematics patterns, while it does not reduce the offsets from the nominal kinematics curves. Overall, the best performance was obtained by the SVD-based pose estimator, while the performance of the PCT pose estimator in its optimal version was not satisfactory. However, the knee kinematics errors reached 5 deg for rotations and 10 mm for translations).

**Conclusion:**

Given the favorable experimental conditions of this study (soft tissue artifacts determined from a young, healthy and non overweight subject), the errors found in estimating the knee kinematics have to be considered unsatisfactory even if the best performing pose estimator is used. Therefore, it is the authors' opinion that the movement analysis research community should make additional efforts in the search of more subject specific error models to increase the accuracy of joint kinematics estimations.

## Introduction

In the last two decades, numerous human movement scientists faced the problem of the in vivo and non-invasive reconstruction of the skeletal movement from measures of the 3D position of points located on the human body surface. To this purpose, the estimation of the pose (position and orientation) of skeletal bones is required. In order to determine the pose of a bone, the definition of a reference frame (RF) attached to it, typically an orthogonal RF defined by bone anatomical landmarks (ALs), is required (anatomical RF). In order to gain more freedom in positioning the markers on the body segment, a technical RF is often identified from the position of the markers attached to the body segment. In this case, the rigid body transformation parameters between the two systems of axes can be obtained with a calibration procedure of the bone ALs [1].

Three sources of errors typically affect the in vivo estimation of the pose of a skeletal bone when non invasive techniques are used: instrumental errors, soft tissues artifacts and AL mislocation. Reviews of such sources of errors and their effect on joint kinematics were recently published [2-5]. Numerous techniques have been proposed to reduce the effect of one or more of these sources of error. Some of them were based on marker attachment devices [6], others were based on computational methods. The latest motion capture technology allows the positioning of a high number of markers on all sides of the surface of each body segment, thus facilitating the use of redundant markers attached directly to the subject skin, which renders in some cases the use of attachment devices obsolete. The same consensus has not been reached yet in the determination of an "optimal" computational method for the determination of the bone pose when the above mentioned sources of error are present.

Skeletal bone pose has been determined from marker positions using various methods including geometrical and optimal. Usually the geometrical pose estimators do not make use of redundant information included in the marker position. Typically, an axis of the RF is defined as the oriented line passing through two of the markers attached to the body segment, a second is defined as the axis perpendicular to the first and lying on the plane identified by the three markers, and the third one is obtained from the cross product of the unit vectors of the two axes already defined. Anatomical RFs are normally identified by the relevant AL positions using geometrical methods with few exceptions [7]. The optimal pose estimators derive the rigid body transformation parameters using least squares methods to solve the difference equation by using either the matrix characteristic equation [8], the singular value decomposition (SVD) [9-13] or iterative methods [14]. In some cases, non-least squares method are used [15].

All the above mentioned pose estimators have pros and cons the importance of which is difficult to determine with in vivo experiments without using invasive techniques. In fact, while the estimation of instrumental errors is not problematic, the in vivo quantification of soft tissue artifacts and their effect on the determination of the bone pose is still an open issue. Recently, several attempts have been made to this respect using various approaches [16,17]. Simulation studies have also been proposed with the limitation of imposing error time histories not generated from experimental observations [18].

Some studies compared the pose estimators mentioned above [13,19]. In the latter study the goal was to test the examined pose estimators' performance in the case of ill-conditioned marker distribution, since some of the optimal pose estimators are very sensitive to highly symmetric spatial distribution of the marker cluster.

Some of the mentioned studies focused their attention on a specific joint, the knee, or a specific segment, the thigh, since soft tissues artifacts have great effects on the determination of the thigh kinematics, which is of particular relevance in gait analysis.

In this study, we evaluated the performance of various pose estimators by implementing a four-bar linkage (FBL) model of the knee attached to rigid models of the tibia end the femur and relevant ALs. The latter bone was equipped with virtual markers the trajectories of which, during simulated FBL movement, were corrupted by both instrumental noise and soft tissue artifacts. By including AL mislocation information in the model, an evaluation of the performance of the selected pose estimators was obtained.

## Methods

**Figure 1:**
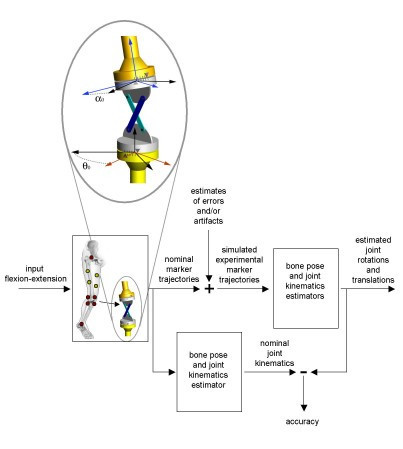
Block diagram of the methods used in the study. A nominal knee kinematics was generated from the position of virtual markers attached to a four bar linkage model of a human knee moved by a knee flexion extension recorded during a gait cycle. The positions of markers on the thigh were then corrupted by instrumental noise and soft tissue artifacts and anatomical landmark mislocation was also included. Pose estimators were then applied to the corrupted virtual marker positions and an estimation of the thigh pose and consequent knee kinematics was obtained. The inset shows the details of the four bar linkage model used in the study.

The methods used for the present study are represented by the diagram in Figure 1. The pattern of a typical knee flexion-extension during a gait cycle was fed into a knee model which generated a six-components knee kinematics (nominal knee kinematics) and relevant thigh marker trajectories. The marker trajectories were corrupted with both instrumental noise and soft tissue artifacts. Then, a selected pose estimator was used followed by the joint kinematics estimator. The estimated knee kinematics was then compared to the nominal kinematics in order to evaluate the performance of the selected pose estimators.

### The knee model

The knee joint was modeled as a four bar linkage [20] (inset Figure 1), a mechanism characterized by a movement driven by a single degree of freedom and by four parameters: Anterior Cruciate Ligament (ACL) and Posterior Cruciate Ligament (PCL) lengths (L_ACL and L_PCL) and ACL-PCL insertion point distances on both femur and tibia (D_femur and D_tibia). The FBL mechanism allowed linear displacements between the femur and the tibia that could be represented as a function of its DoF (input knee flexion-extension). The model included two additional parameters: a rotation angle about the femur longitudinal axis α_0 _(*FBL rotation offset*) used to introduce an ab-adduction angle in the model kinematics, and a rotation angle θ_0 _(*tibia rotation offset*) around the tibia longitudinal axis taking into account the internal external rotation angle between femur and tibia in upright posture. The values originally set for the knee model parameters are reported in Table 1 and matched those proposed by Gill and O'Connor [20]. The values for α_0 _and θ_0 _were set based on the subject's knee kinematics and upright posture knee alignment, respectively.

**Table 1:** Parameters of the four bar linkage model of the knee.

*FBL parameters [mm]*				*knee parameters [deg]*	
*L_ACL*	*L_PCL*	*D_femur*	*D_tibia*	*α*_0_	*θ*_0_
29.9	32.2	12.7	30.5	10	15

### The anthropometric and kinematic data

**Figure 2:**
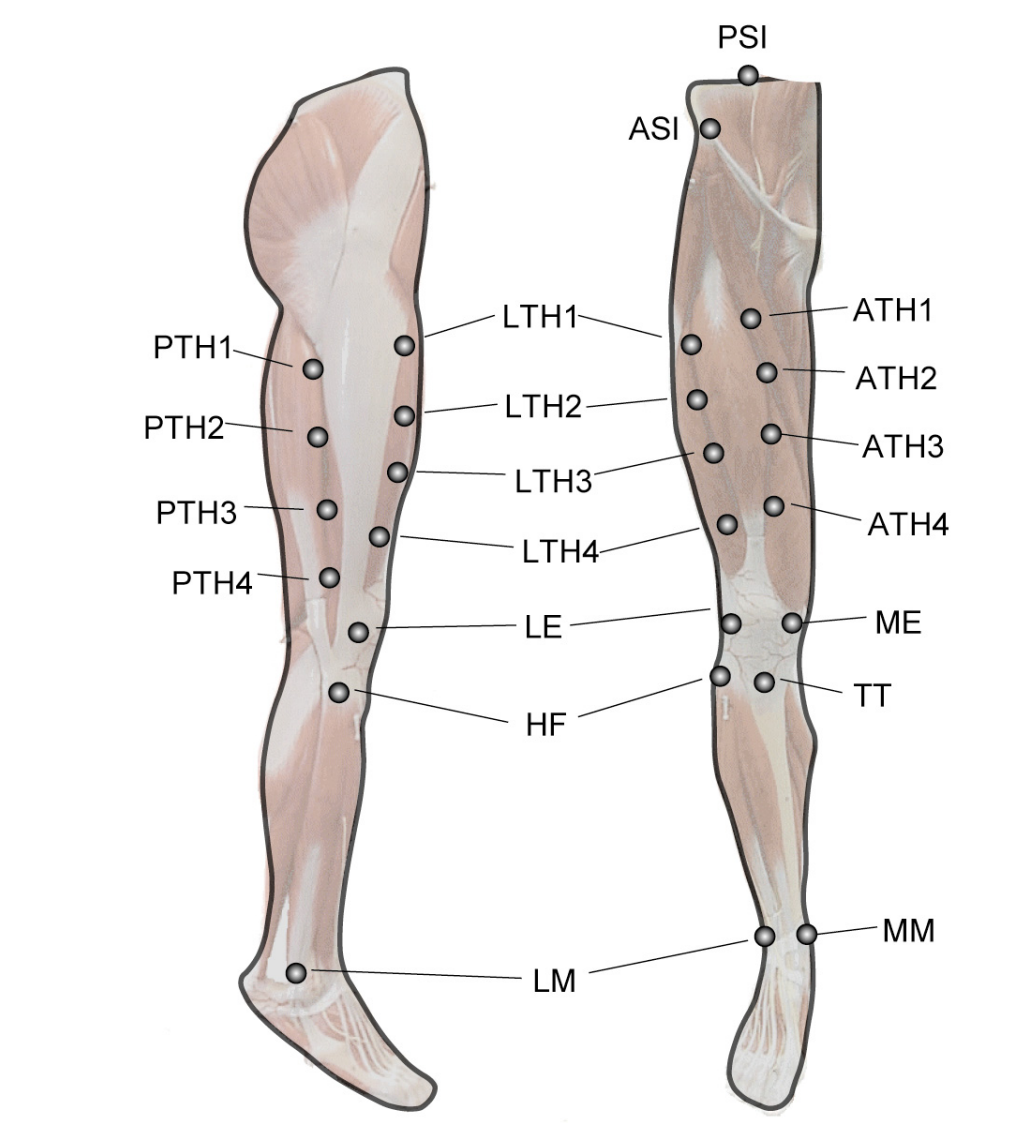
Locations and names of the selected twelve thigh markers and the thigh and shank anatomical landmarks.

The two segments forming the knee joint of a healthy male subject (1.8 m, 70 kg), thigh and shank, were modeled as follows. Twelve markers were attached to the subject's thigh. Additional markers were positioned over the lateral and medial femur epicondyles and over the tibia ALs. (Figure 2). The position of all markers was acquired during an upright posture static trial using a 6-camera VICON motion capture system. The femur head position was determined using an *ad hoc *experiment [21]. The FBL knee model was made to move by the imposed knee flexion-extension angle pattern. The FBL knee model kinematics was used as nominal kinematics. The thigh and shank models were then attached to the FBL model and the simulated kinematics of all markers and ALs were then generated.

### Generation of noisy movement data

**Figure 3:**
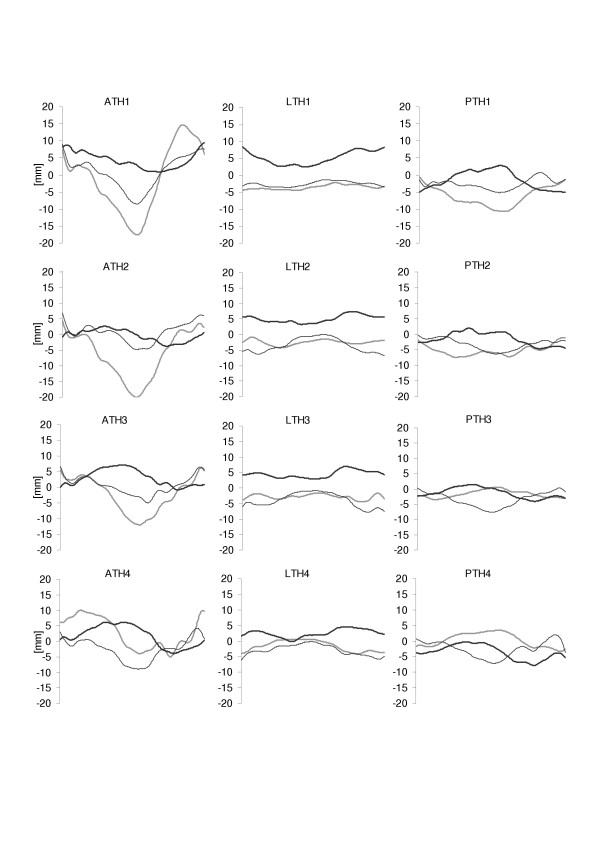
Patterns of the soft tissue artifacts affecting the position of the twelve thigh markers during a gait cycle performed by the analyzed subject in the femoral anatomical frame. X (positive direction: anterior): thick gray lines; Y (positive direction: proximal): thick black lines; Z (positive direction: from left to right): thin black lines.

Noisy marker trajectories were generated by adding two different types of noise to the twelve noise-free marker trajectories obtained for the thigh markers from the FBL knee nominal kinematics. First, instrumental noise was added to the marker trajectories and then, soft tissue artifacts were added. The instrumental noise had a zero mean normal distribution with 1 mm standard deviation. The soft tissue artifacts were modeled according to those obtained by Camomilla et al. [16]. Twelve different artifacts relative to the twelve different marker locations were used (patterns are reported in Figure 3 and mean and standard deviations with respect to upright posture configuration in Table 2). Two different marker configurations were used for the analysis. One was made up from the markers ATH1, ATH2, LTH3 and PTH1, the other used all the twelve markers available.

**Table 2:** Mean and standard deviation values of the soft tissue artifacts expressed in the femoral anatomical frame applied to the nominal positions of the twelve thigh markers. Data regarding the markers used in the 4-marker configuration are in bold. X positive direction: anterior; Y positive direction: proximal; Z positive direction: from left to right.

*marker*		*1*			*2*			*3*			*4*	
*[mm]*	*X*	*Y*	*Z*	*X*	*Y*	*Z*	*X*	*Y*	*Z*	*X*	*Y*	*Z*
*ATH*	**-1.7**	**3.9**	**0.2**	-7.7	-0.8	-0.2	**-3.0**	**3.3**	**0.1**	2.3	1.8	-3.0
	**(10.0)**	**(2.2)**	**(4.5)**	(7.3)	(2.0)	(2.8)	**(5.4)**	**(2.7)**	**(2.5)**	(5.1)	(3.5)	(3.5)
*LTH*	-3.5	4.8	-2.5	-2.6	5.0	-3.2	**-2.8**	**4.4**	**-3.7**	-1.4	2.6	-2.6
	(0.7)	(1.9)	(0.7)	(0.9)	(1.3)	(2.2)	**(0.8)**	**(1.2)**	**(2.2)**	(1.7)	(1.3)	(1.6)
*PTH*	**-6.7**	**-1.1**	**-2.7**	-5.6	-1.2	-3.4	-1.6	-1.2	-3.8	0.6	-3.5	-2.7
	**(2.6)**	**(2.6)**	**(1.5)**	(1.4)	(2.0)	(1.8)	(1.2)	(1.7)	(2.3)	(2.2)	(2.4)	(2.5)

In order to quantify the combined influence on the estimated kinematics of instrumental noise, soft tissue artifacts and AL mislocation, the medio-lateral axis of the femur anatomical RF (the same used by Grood and Suntay [22] for representing knee flexion-extension) was made to rotate ± 5 deg about the femur long axis.

### Bone pose estimators

Once the two sets of thigh marker trajectories containing (a) instrumental noise and (b) instrumental noise added to soft tissue artifacts were generated, four different bone pose estimators were applied to them so that the pose of a technical RF could be determined. Thus, the anatomical RF pose could be estimated using the information obtained by the AL calibration. Finally, the knee model kinematics could be determined using the method proposed by Grood and Suntay [22] and compared to the nominal knee kinematics.

The following four bone pose estimators were tested in this study.

A geometrical method using the selected four markers. The origin of the technical frame was located at the midpoint of the line joining ATH2 and LTH3. The direction of the line joining the origin and ATH1 pointing at ATH1 was projected on the plane formed by ATH2, LTH3 and PTH1 defined the Y axis. The axis perpendicular to the Y-axis and lying on the plane formed by ATH2, LTH3 and PTH1 pointing to the right was used as the Z-axis. The X-axis was obtained from the cross product of the unit vectors of the Y-axis and the Z-axis.

The SVD-based pose estimator. The SVD-based pose estimator finds the pose of a reference marker configuration (typically obtained in static conditions) that minimizes the elastic energy of identical virtual springs connecting the corrupted marker locations (recorded during the movement) to the relevant marker location in the reference configuration. The SVD-based pose estimator was implemented as in Cappozzo et al. [12].

The Pointer Cluster Technique (PCT) based on the identification of the marker cluster tensor of inertia [15]. The PCT pose estimator consists of two steps: first, a single mass value (typically a unit value) is assigned to all markers forming the cluster attached to a body segment. In every instant *i *during the movement the tensor of inertia of the configuration D_*i *_of the corrupted marker locations is generated and the pose of the RF formed by the principal axes of inertia of D_*i *_is determined (PCTu). Secondly, the marker mass values are adjusted until the difference between the sum Λ_*i *_of the square values of the tensor of inertia eigenvalues obtained from the marker cluster during movement and the sum Λ_0 _resulting from a reference marker configuration D_0_, is minimum (PCTa). Both steps were implemented. The PCTa objective function was minimized using the function "lsqnonlin" (Gauss-Newton method) available in Matlab (MathWorks inc.). As suggested by the authors, the termination tolerance of the function value was set equal to 0.1. Occasionally, both PCTu and PCTa pose estimators produced instantaneous orientation inversions of the principal axes that were removed by comparing axes orientation in adjacent time instants.

## Results and discussion

**Figure 4:**
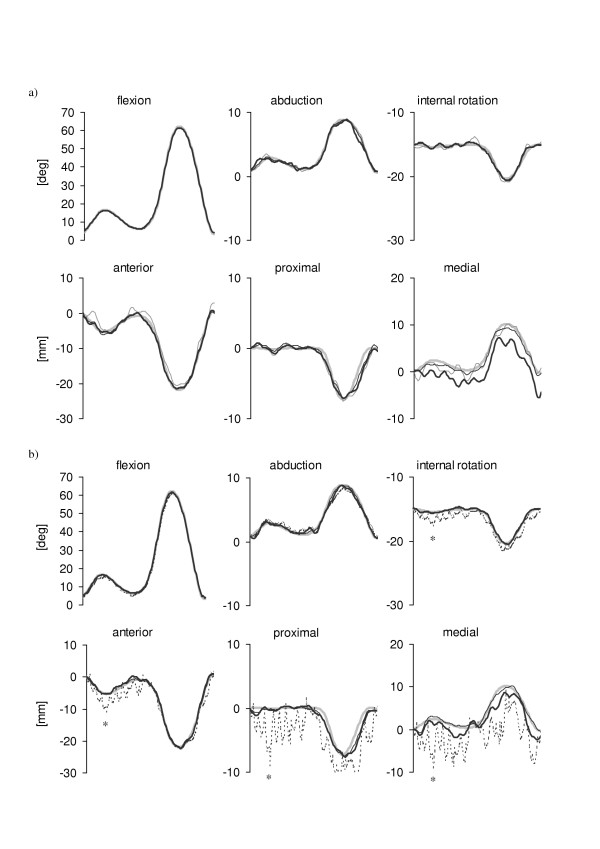
Patterns of the six knee kinematics components obtained after applying the selected pose estimators to a 4-marker cluster (a) and to a 12-marker cluster (b) when only instrumental errors were added to the marker nominal positions. Nominal patterns: thick gray lines; estimates from the geometric pose estimator: thin gray lines; estimates from the SVD-based pose estimator: thin black lines; estimates from the PCTu pose estimator: thick black lines; estimates from PCTa pose estimator: dotted black lines.

The six components of the nominal knee kinematics are illustrated in Figure 4, superimposed to the knee kinematics estimated using the four methods applied to marker trajectories affected by the instrumental noise (4-marker cluster Figure 4a, 12-marker cluster Figure 4b). The knee kinematics obtained by applying the PCTa pose estimator is not shown for 4-marker configurations since the minimization was not successful due to the reduced number of markers used in the algorithm. All tested pose estimators provided satisfactory knee kinematics estimates for both rotational and translational components.

The 12-marker configuration prevented the use of a geometric estimator and allowed a slight improvement of the knee kinematics estimation (PCTu and SVD-based), more evident in the two minor rotational components (ab-adduction and internal-external rotation). However, since the difference between the kinematic patterns obtained from each of the various pose estimators and the nominal kinematics were limited for both rotations and translations (< 2 deg and < 4 mm, respectively), the effects of the instrumental noise could be considered as less important. Therefore, when soft tissue artifacts are absent (as in the case of the gait analysis of subjects with prostheses and/or orthoses), in most cases it is not necessary to use more than three or four markers and a geometrical pose estimator can be satisfactory. Knee kinematics PCTa estimates presented irregular patterns. This undesired effect is due to the fact that in each instant of time the optimization function used in the PCTa, instead of operating on each eigenvalue of the tensor of inertia of the marker configuration, minimizes the difference between Λ and Λ_0_, the sums of the squares of the three eigenvalues. As a consequence, the proportions among the eigenvalues values can vary (and therefore the relevant eigenvectors) keeping the sum Λ very close to Λ_0_. The frame of the plots in Figure 4b marked with a star (*) is an example of such malfunctioning. The eigenvalues of the reference configuration D_0 _were 50190 mm^2^, 81340 mm^2 ^and 113790 mm^2^, those of the instantaneous tensor of inertia of the configuration D_1 _minimizing the optimization function at the first frame were 48690 mm^2^, 82380 mm^2 ^and 113700 mm^2^, and those obtained at the selected frame (D*) were 40410 mm^2^, 87450 mm^2 ^and 113150 mm^2^, respectively. Whereas the eigenvalues of D_1 _were close to those of D_0_, the eigenvalues of D* were remarkably different and so were the relevant eigenvectors. Therefore, the axes of inertia of the marker configuration at the selected frame were different from the reference ones. Such difference reflected on the low accuracy of the knee kinematics estimate.

**Figure 5:**
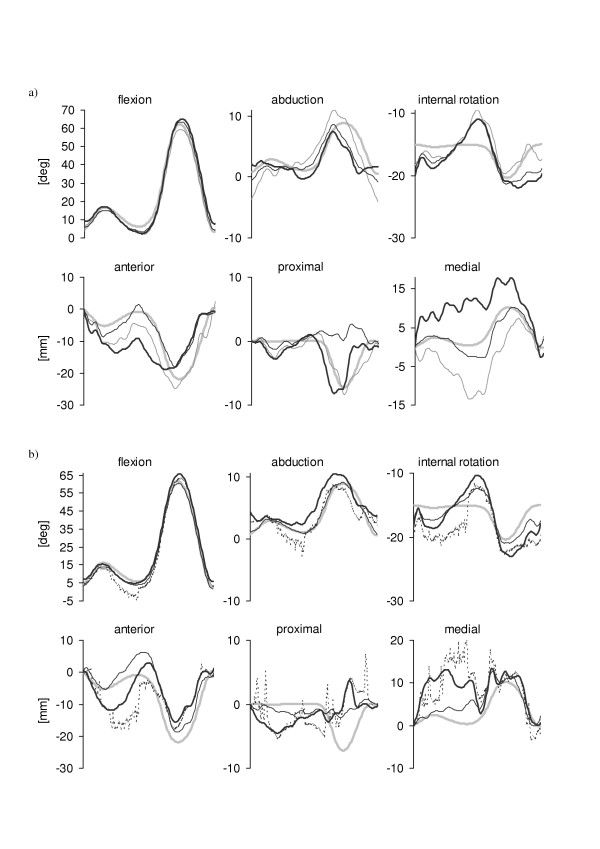
Patterns of the six knee kinematics components obtained after applying the selected pose estimators to a 4-marker cluster (a) and to a 12-marker cluster (b) when both instrumental errors and soft tissue artifacts were added to the marker nominal positions. See caption of Figure 4 for legend details.

Figure 5 illustrates the knee kinematics obtained in the case of soft tissue artifacts added to the instrumental noise. The mean and standard deviation values of the differences between each pose estimator and the nominal pattern are reported in Table 3. A large mean value of such difference highlights the presence of an offset in the relevant curve, whereas a large standard deviation value highlights the pattern dynamics discrepancy of the same curve. In the case of the 4-marker configuration the geometric pose estimator overestimated the range of variability of the two minor angle components and underestimated the knee flexion-extension. The errors in the estimation of the knee translations were in some cases over 10 mm. Overall, the PCTu estimator did not perform better than the geometric estimator while the SVD-based pose estimator showed an overall improvement with respect to the geometric estimator. The pattern dynamics of the SVD-based pose estimates were generally closer to the nominal ones than those obtained using the PCTu pose estimator (see relevant values in Table 3). In the 12-marker configuration case, the SVD-based pose estimator showed an overall higher accuracy than both PCT pose estimators. However, as confirmed by the values in Table 3, the improvement with respect to the 4-marker configuration is not consistent in all components. Therefore, the use of 12-marker configurations seems to be only partially justified. However, it is reasonable to expect that for subjects with larger soft tissue artifacts than those of the subject considered in this study, the 12-marker configuration could provide more accurate pose estimates.

**Table 3:** Mean and standard deviation values of the error patterns of the knee kinematics reported in Figure 5.

*pose estimator*	*flexion [deg]*	*abduction [deg]*	*internal [deg]*	*anterior [mm]*	*proximal [mm]*	*medial [mm]*
*geometric*	-2.3 (2.0)	-0.3 (2.4)	0.6 (2.3)	-3.6 (2.8)	-0.6 (1.0)	-6.5 (4.5)
*SVD 4-markers*	-0.6 (2.2)	-1.0 (1.8)	-0.7 (2.4)	1.1 (3.6)	1.9 (3.0)	-0.8 (1.8)
*SVD 12-markers*	-1.7 (0.7)	0.1 (0.4)	-0.4 (1.7)	3.4 (2.4)	0.6 (2.3)	1.8 (2.0)
*PCTu 4-markers*	0.5 (3.6)	-1.3 (1.9)	-1.1 (2.9)	-3.2 (7.1)	-0.5 (1.9)	6.7 (3.4)
*PCTu 12-markers*	1.0 (2.4)	1.5 (1.3)	-0.8 (2.9)	1.5 (5.6)	-0.2 (3.8)	5.1 (3.9)
*PCTa 12-markers*	-2.3 (3.7)	-0.2 (1.7)	-2.5 (2.8)	-1.2 (7.4)	0.2 (4.3)	5.6 (6.1)

**Figure 6:**
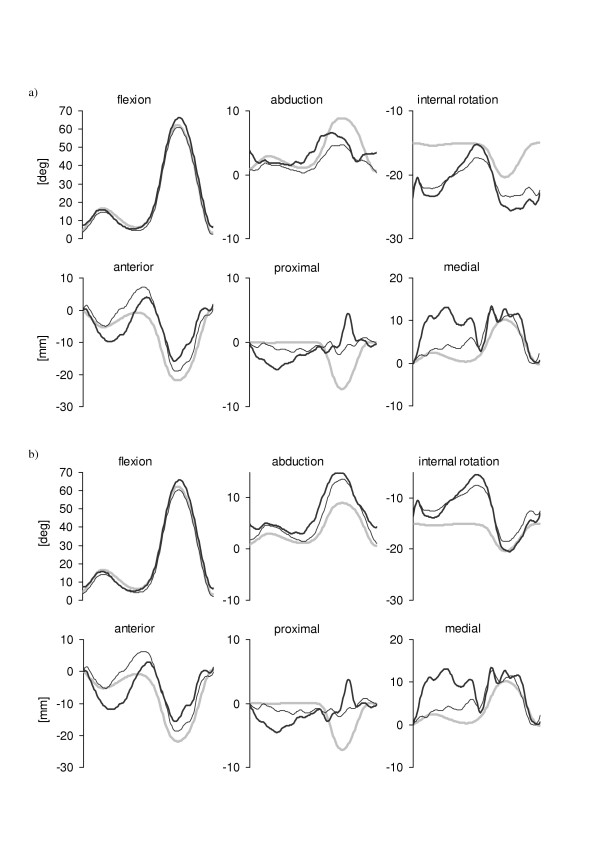
Patterns of the six knee kinematics components obtained after applying the selected pose estimators to the 12-marker cluster when instrumental errors and soft tissue artifacts and were added to the marker nominal positions and the femur anatomical reference frame was made to rotate internally and externally as the effect of AL mislocations (a: +5 deg, b: -5 deg). See caption of Figure 4 for legend details. PCTa estimated patterns are not included.

The effects of the femur internal-external rotations (± 5 deg) due to AL mislocation combined to both instrumental noise and soft tissue artifacts on the performance of the pose estimators, are reported in Figure 6 for the 12-marker configuration. Results showed that even a reduced error in determining the orientation of the femur anatomical axes added to the soft tissue artifacts can introduce remarkable errors in determining the knee kinematics (Table 4). The performance of SVD-based pose estimator was clearly better than that of the PCT pose estimator, even in the presence of AL mislocations.

**Table 4:** Mean and standard deviation values of the error patterns reported in Figure 6.

*pose estimator*	*flexion [deg]*	*abduction [deg]*	*internal [deg]*	*anterior [mm]*	*proximal [mm]*	*medial [mm]*
*SVD (+5 deg)*	1.6 (0.7)	1.7 (1.5)	4.7 (1.8)	3.8 (2.5)	1.8 (1.8)	1.6 (1.3)
*PCTu (+5 deg)*	2.1 (1.6)	1.7 (1.3)	5.2 (2.8)	4.8 (2.6)	3.1 (2.6)	5.0 (4.3)
*SVD (-5 deg)*	2.0 (0.7)	1.9 (1.3)	4.0 (1.9)	3.5 (2.3)	1.8 (1.6)	2.1 (1.7)
*PCTu (-5 deg)*	1.9 (1.5)	3.3 (1.6)	3.6 (3.1)	5.1 (2.7)	3.0 (2.3)	5.2 (3.8)

The study focused on the knee kinematics estimation from noisy thigh data. The shank pose estimation was considered noise-free for two reasons: a) results interpretation is simpler when the joint kinematics error contributions are presented separately for the two segments forming the joint and b) the errors affecting the thigh pose estimation are typically higher than those affecting the shank pose estimation (larger thigh soft tissue artifacts and higher risk of thigh AL mislocation).

The soft tissue artifacts patterns used in this study were obtained from a young healthy, non overweight subject. For subjects with different characteristics, soft tissue artifacts could be more disruptive, therefore the errors shown in this study may represent an estimate of a minimum possible error.

## Conclusion

The present study was carried out to report the level of accuracy that can be reached in estimating bone pose and joint kinematics by some bone pose estimator methods selected from those proposed in the last two decades. The study took into account all sources of errors typically affecting joint kinematics estimation. By focusing on the knee kinematics estimated from corrupted thigh marker trajectories, this study highlighted the advantages and the limitations of both simple and sophisticated pose estimators. Overall, the SVD-based pose estimator showed the best performance. However, errors up to 5 degrees for rotations and 10 mm for translations were found even in the most favorable conditions. Therefore, since a large inter-subject variability of thigh soft tissue artifacts is commonly recognized, it is the authors' opinion that future research should aim at using more subject specific data to correct the effects of both the soft tissue artifacts and AL mislocations.

## References

[B1] Cappozzo A, Catani F, Della Croce U, Leardini A (1995). Position and orientation in space of bones during movement: anatomical frame definition and determination. Clin Biomech (Bristol, Avon).

[B2] Cappozzo A, Della Croce U, Leardini A, Chiari L (2005). Human movement analysis using stereophotogrammetry. Part 1: theoretical background. Gait Posture.

[B3] Chiari L, Della Croce U, Leardini A, Cappozzo A (2005). Human movement analysis using stereophotogrammetry. Part 2: instrumental errors. Gait Posture.

[B4] Leardini A, Chiari L, Della Croce U, Cappozzo A (2005). Human movement analysis using stereophotogrammetry. Part 3. Soft tissue artifact assessment and compensation. Gait Posture.

[B5] Della Croce U, Leardini A, Chiari L, Cappozzo A (2005). Human movement analysis using stereophotogrammetry. Part 4: assessment of anatomical landmark misplacement and its effects on joint kinematics. Gait Posture.

[B6] Cappozzo A, Catani F, Leardini A, Benedetti MG, Della Croce U (1996). Position and orientation in space of bones during movement: experimental artefacts. Clin Biomech (Bristol, Avon).

[B7] Della Croce U, Camomilla V, Leardini A, Cappozzo A (2003). Femoral anatomical frame: assessment of various definitions. Med Eng Phys.

[B8] Veldpaus FE, Woltring HJ, Dortmans LJ (1988). A least-squares algorithm for the equiform transformation from spatial marker co-ordinates. Journal of Biomechanics.

[B9] Arun KS, Huang TS, Blostein SD (1987). Least-Squares Fitting of 2 3-D Point Sets. IEEE Trans Pattern Anal Mach Intell.

[B10] Soderkvist I, Wedin PA (1993). Determining the movements of the skeleton using well-configured markers. Journal of Biomechanics.

[B11] Challis JH (1995). An examination of procedures for determining body segment attitude and position from noisy biomechanical data. Medical Engineering & Physics.

[B12] Cappozzo A, Cappello A, Della Croce U, Pensalfini F (1997). Surface-marker cluster design criteria for 3-D bone movement reconstruction. IEEE Trans Biomed Eng.

[B13] Cheze L, Fregly BJ, Dimnet J (1995). A solidification procedure to facilitate kinematic analyses based on video system data. Journal of Biomechanics.

[B14] Cappello A, La Palombara PF, Leardini A (1996). Optimization and smoothing techniques in movement analysis. Int J Bio-Med Comput.

[B15] Andriacchi TP, Alexander EJ, Toney MK, Dyrby C, Sum J (1998). A point cluster method for in vivo motion analysis: applied to a study of knee kinematics. Journal of Biomechanical Engineering.

[B16] Camomilla V, Donati M, Cappozzo A (2005). Non-invasive soft tissue artefact assessment: September 5-7th 2005; University of Salford, Salford..

[B17] Cappello A, Stagni R, Fantozzi S, Leardini A (2005). Soft tissue artifact compensation in knee kinematics by double anatomical landmark calibration: performance of a novel method during selected motor tasks. IEEE Trans Biomed Eng.

[B18] Alexander EJ, Andriacchi TP (2001). Correcting for deformation in skin-based marker systems. Journal of Biomechanics.

[B19] Carman AB, Milburn PD (2005). Determining rigid body transformation parameters from ill-conditioned spatial marker co-ordinates. Journal of Biomechanics.

[B20] Gill HS, O'Connor JJ (1996). Biarticulating two-dimensional computer model of the human patellofemoral joint. Clinical Biomechanics.

[B21] Camomilla V, Cereatti A, Vannozzi G, Cappozzo A (2005). An optimized protocol for hip joint centre determination using the functional method. J Biomech.

[B22] Grood ES, Suntay WJ (1983). A joint coordinate system for the clinical description of three-dimensional motions: application to the knee. Journal of Biomechanical Engineering.

